# Restraint stress prolongs Diestrus phase of mouse Estrous cycle

**DOI:** 10.1093/oons/kvag002

**Published:** 2026-03-18

**Authors:** Gwendolyn R Urbain, Alex D Chapman, Brooke M Van Loh, Joseph K Folger, Geoffroy Laumet

**Affiliations:** Department of Physiology, Michigan State University, East Lansing, MI, United States; Department of Physiology, Michigan State University, East Lansing, MI, United States; Neuroscience Graduate Program, Michigan State University, East Lansing, MI, United States; Neuroscience Graduate Program, Michigan State University, East Lansing, MI, United States; Department of Animal Science, Michigan State University, East Lansing, MI, United States; Department of Physiology, Michigan State University, East Lansing, MI, United States; Department of Physiology, Michigan State University, East Lansing, MI, United States; Neuroscience Graduate Program, Michigan State University, East Lansing, MI, United States

**Keywords:** Stress, estrous cycle, mouse, female reproductive health

## Abstract

Globally, stress levels among women of reproductive age are rising, while fertility rates continue to decline. Despite this correlation, a causal link between stress and reduced fertility remains unclear. Experimental studies have shown that severe chronic stress can disrupt reproductive function, but the effects of mild stress, more representative of the daily stress experienced by most women, are still poorly understood. This study aims to identify how mild stress affects the mouse estrous cycle. Nineteen mice were vaginally lavaged daily one week before stress, during 3-day stress, and one week after stress. The mild stress paradigm consisted of two hours of repeated restraint stress each day for three days. Restraint stress disrupted the estrous cycle causing a longer cycle length in stressed mice, characterized by an extended duration in the diestrus phase. These findings suggest that even moderate stress perturbs normal reproductive cycling, potentially contributing to reduced fertility. This work highlights the need to further explore how everyday stressors may subtly impair reproductive health.

## Introduction

Fertility rates have been decreasing for the past decade (Driscoll and Hamilton 2025). At the same time, women are reporting daily stress at an increasing rate (APA 2023). Stress impacts women at a higher rate than men, a trend that might result from the dynamic neurochemical fluctuations in the female brain driven by cyclical hormonal changes (Lovick 2012; Thiyagarajan et al. 2025). By using an animal model to study female cycling, external variables are controlled and eliminated such as biases/expectations about how stress impacts the body, patient, and psychological history (Caligioni, 2009). Rodents are commonly used to model the estrous cycle as their shorter reproductive cycle provides a quicker way to investigate changes, and estrous cycle tracking can serve as a proxy for the hormonal state of female mice (Rocks et al. 2022). Previously, chronic stress experiments have involved female mice being stressed over 30 consecutive days through cold water exposure, which caused decreased reproductive function (Casillas et al. 2021). Additionally, severe stress such as exposure to predator odors or 5-hours of restraint stress for 31 days caused an impaired luteinizing hormone (LH) surge, preventing ovulation from occurring (Wagenmaker and Moenter, 2017). However, these extreme paradigms trigger life-threatening stress and may not appropriately model the daily stress experienced by average women. Therefore, there is an urgent need to address whether mild stress impacts the estrous cycle. In this project, a well-validated model of repeated restraint stress was used to mimic daily stress (Avona et al. 2020). We hypothesized that acute restraint stress would dysregulate the estrous cycle.

## Materials and methods

### Animals

All animal procedures were approved by Michigan State University (MSU) Institutional Animal Care and Use Committee (IACUC) and in accordance with the National Institutes of Health (NIH) Guide for the Care and Use of Laboratory Animals. The breeder mice were originally obtained from The Jackson Laboratory (JAX#000664) and subsequently bred in our laboratory colony. All animals were housed under a 12-hour light–dark cycle with *ad libitum* water and feeding. C57Bl/6 J adult female (15 weeks old) mice were used as control (n = 9) or stress (n = 10) conditions. To minimize potential litter effects, each experimental cage consisted of animals from a single litter (3–5 female mice per cage). Cages were randomly assigned to the control condition or to the stress condition. Animals remained group-housed throughout the experiment, except during the restraint stress sessions, when mice were individually restrained in conical tubes.

### Vaginal lavaging

Mice were vaginally lavaged to collect vaginal epithelial cells by pipetting fifteen microliters of water onto the vaginal surface and removing the water. This process was repeated four times to ensure cell collection. The cells were then spread onto a slide in a circular fashion for even distribution.

### Staining and staging

**Figure 1:**
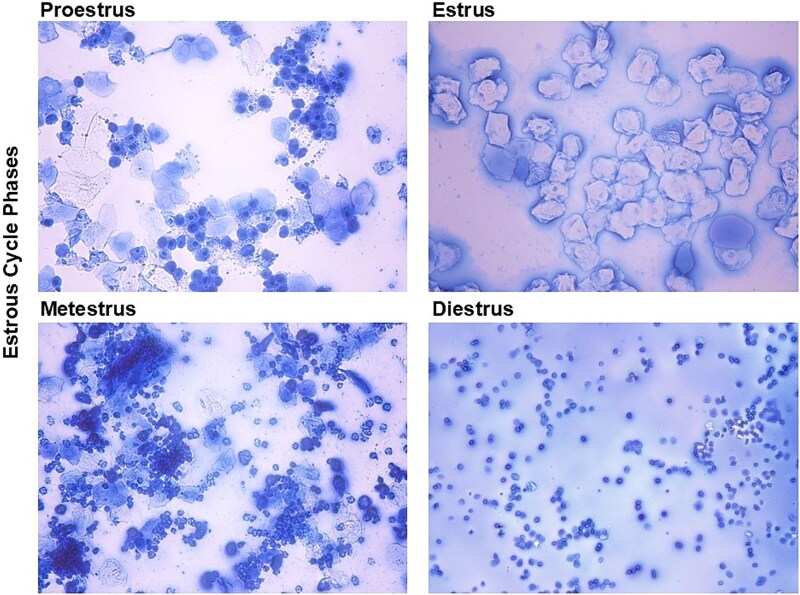
Estrous cycle phase determined by vaginal smears stained with 0.5% Evans blue and staged using brightfield microscopy. **Proestrus**: Predominantly nucleated epithelial cells, indicating the onset of follicular development. **Estrus**: Abundant cornified (anucleate) epithelial cells, reflecting peak estrogen levels and sexual receptivity. **Metestrus**: Mixed cell population including leukocytes, nucleated, and cornified epithelial cells, marking the transition post-ovulation. **Diestrus**: Predominantly leukocytes, indicating low estradiol levels and luteal phase dominance

The slides with vaginal cells were stained using 0.5% Evans Blue dye and staged according to vaginal smear cytology rules (Cora et al. 2015). The smears consist of four phases: Proestrus (follicular phase), Estrus (ovulation), Metestrus (initial part of luteal phase), and Diestrus (later part of luteal phase) as illustrated in Fig. 1. The mouse completed a full cycle by going through each phase in order.

### Restraint stress

Mice were placed into 50 mL conical tubes modified for restraint with breathing holes, and crumpled paper towels were inserted behind the mouse to hold it in place. Mice were restrained from 1100 h to 1300 h daily for 3 days.

### Timeline

**Figure 2:**
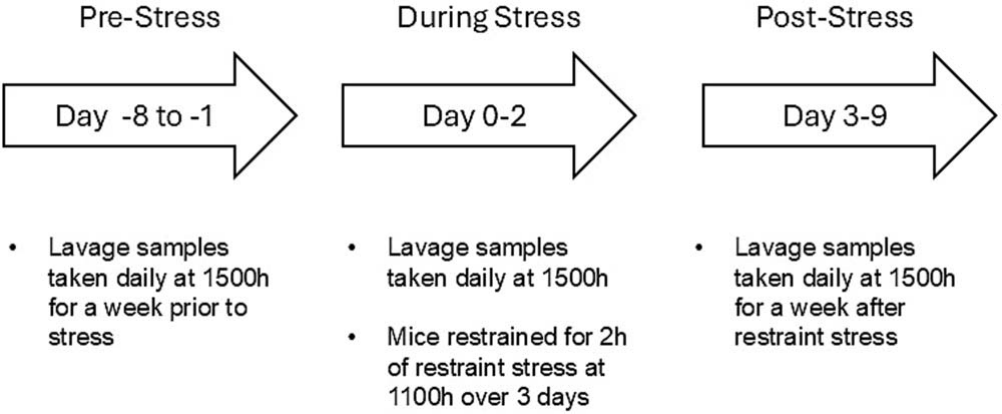
Experimental timeline of the 17 days consisting of pre-stress (days −8 to −1), during stress (days 0–2), and post-stress (days 3–9) periods

The mice were lavaged daily at 1500 h for the entire 17-day period. This consisted of a pre-stress, during-stress, and post-stress timespan. The pre-stress phase consisted of seven days before stress to determine if the mice were normally cycling before stress. During the stress period, control animals had no interactions with experimenters and remained undisturbed in their home cages with their cagemates in the housing room. Stress mice were restrained for two hours at 1100 h for three days. The post-stress period began at the end of 3 days of restrain stress and lasted for seven days. This timeline is represented in Fig. 2.

### Identification of cycles

After staging slides for each mouse, each phase was assigned a number (Proestrus = 1, Estrus = 2, Metestrus = 3, Diestrus = 4) for quantification and graphical representation (Table 1). A mouse was classified as cyclic if it completed a full estrous cycle by progressing through all four stages (proestrus, estrus, metestrus, diestrus) in the correct order at least once during the observation period,that repeated stages were permitted, and that cycle counting began on the first day of observation (going through each phase in order i.e. 1 → 2 → 3 → 4, or 2 → 3 → 4 → 1, or 1 → 2 → 2 → 3 → 3 → 4 etc.). Accordingly, sequences such as 2 → 3 → 4 → 2 → 3 → 4 → 4 → 1 were still considered cyclic, as all stages were observed in the appropriate order without reversal. The number of cycles each mouse went through were then counted and put onto a separate table. Mice that did not complete a cycle during the post-stress period were assigned a cycle length of 10 days as 10 days was the maximum observation window. In Table 1, we presented examples of identified cycles in representative female mice.

**Table 1:** Representation of identified cycles in control mice. F1, F2, F3, and F4 represent females 1–4. For each day, the phase each mouse was in is identified and assigned a number (1–4 corresponding with estrous phase). A full cycle is marked by horizontal lines

Date	F1	F2	F3	F4
11-Nov	2	3	3	4
12-Nov	3	4	3	1
13-Nov	4	1	3	2
14-Nov	2	2	4	3
15-Nov	3	2	1	3
16-Nov	4	3	2	4
17-Nov	4	4	3	4
18-Nov	1	4	3	2
19-Nov	2	1	1	2
20-Nov	3	1	1	3
21-Nov	4	2	2	4
22-Nov	1	2	3	1
23-Nov	2	3	4	2
24-Nov	3	3	1	3
25-Nov	3	4	2	4
26-Nov	4	1	3	4
27-Nov	1	2	3	1

### Statistical analysis

The data was analyzed using GraphPad Prism v9. Based on experimental design, unpaired t-test, Fisher’s Exact test, or two-way ANOVA with correction for multiple comparisons were run to determine statistical difference between tested groups.

## Results

### Effect of restraint stress on completion of the Estrous cycle

**Figure 3:**
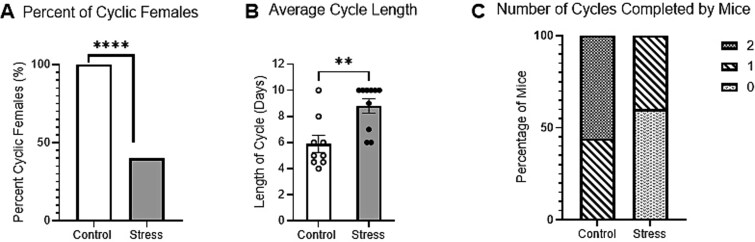
Restraint stress increases cycle length and decreases Total cycles completed. A: An unpaired t-test was used to compare control and stress conditions. Mice were lavaged for 3 days of restraint stress and 7 days after stress (n = 9 ctrl and 10 stress). Fisher’s exact test (^****^P < 0.0001) B: Average cycle length was significantly increased in stress as compared to control. The stress condition combines 3 days of restrain stress and 7 days of post-stress spanning 10 days. Mice that did not complete a cycle during the post-stress period were assigned a cycle length of 10 days. Unpaired t-test (F(8,9) = 1.24, ^**^P < 0.01). C: Restraint stress reduced the number of completed cycles over 10 days compared with stress and control conditions. Fisher’s exact test (^***^P < 0.001).

All mice cycled normally for the 7-day pre-stress phase, before the start of our stress paradigm. As expected, in the control, 100% of female mice remained cyclic during the testing period (Fig. 3A). Restraint stress significantly reduced the number of completed cycles compared to control mice (Fig. 3A). Only 40% of stressed mice remained cyclic (Fig. 3A). Given that fewer stressed mice completed a full cycle, potential impact of stress on the length of the cycle was examined. The control mice had an average cycle length of 5.9 days while stressed mice had an average cycle length of 8.8 days (Fig. 3B). The average length of the estrous cycle was significantly longer in the stress condition as compared to the control (Fig. 3B). Additionally, 56% of the control group completed 2 full cycles, with the remaining 44% completing 1 full cycle over 10 days (Fig. 3C). 60% of stressed mice did not complete a full cycle over the 10-day period (Fig. 3C), and the others completed only 1 cycle (Fig. 3C).

### Restraint stress causes increased time spent in Diestrus phase

**Figure 4:**
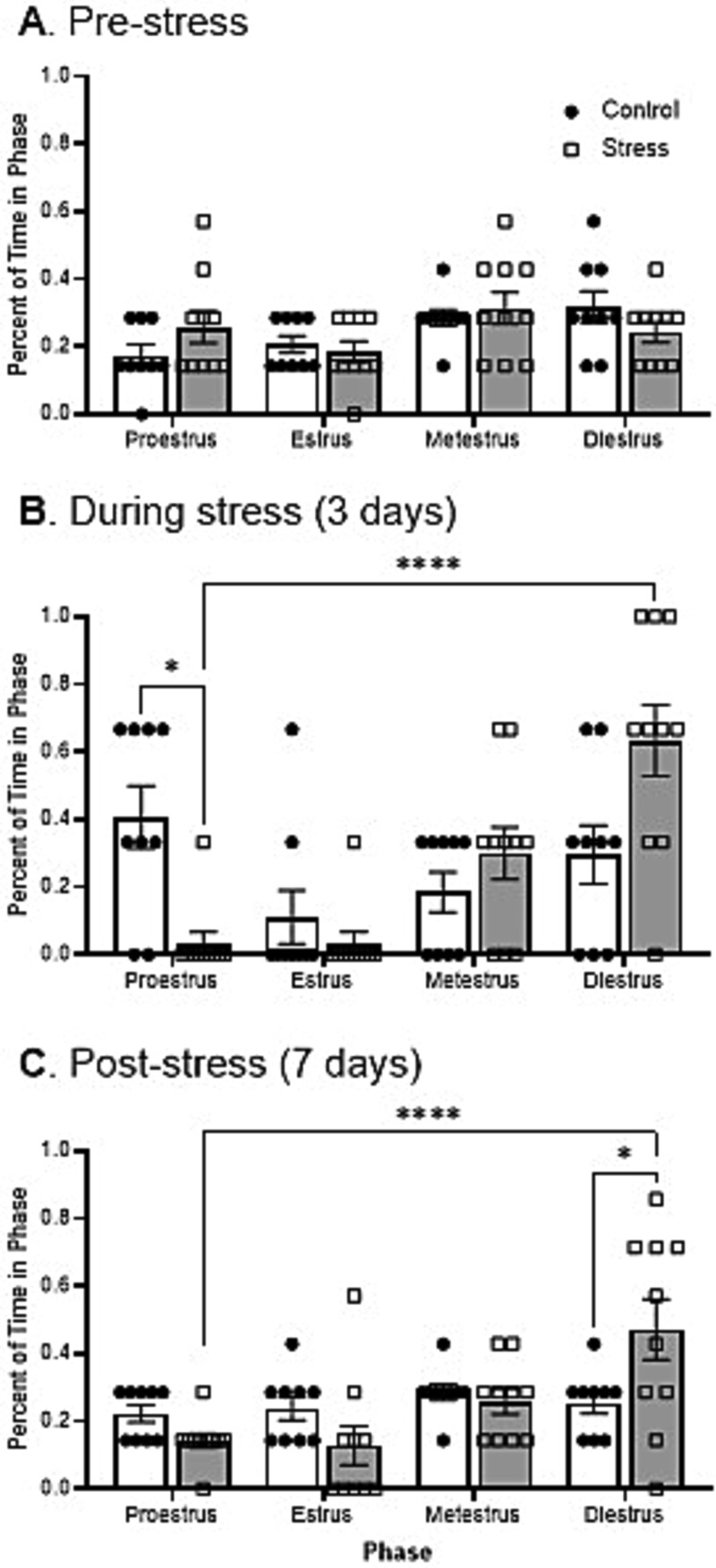
Restraint stress increased the percentage of time spent in diestrus phase and reduced the time in other estrous phases in mice. Pre (A), during (B), and post (C) stress. Two-way ANOVA tests corrected by Tukey’s for multiple test were used to determine the difference between stress and control conditions as well as within each condition’s phases. Two-way ANOVA followed by Tukey’s correction for multiple tests: 6A: F(1,68) = 0.023; 6B: F(1,68) = 3.4e-19, F(3,68) = 9.43; 6C: F(1,68) = 2.01e-17, F(3,68) = 6.63, ^*^P < 0.05, ^**^P < 0.01, ^***^P < 0.001, ^****^P < 0.0001).

As the stressed female mice completed fewer cycles and had longer cycle duration, next the effect of stress on each phase was assessed. To determine whether a difference in phase length was present between stress and control mice, we measured the percentage of time spent in each phase. The pre-stress period showed all mice cycling normally with no difference in time spent in each phase between the control and stress mice (Fig. 4A). In control mice, the times spent in each phase stayed similar through the experiment (Fig. 4, full statistics in Table 2). During the stress period, a significant decrease in time spent in the proestrus phase was seen in the stressed mice compared to control (Fig. 4B). To assess whether stress was influencing a particular phase of the estrous cycle, we compared the time spent in each phase within groups. Stressed mice had a significant increase in time spent in the diestrus phase compared to all other phases during the stress period (Fig. 4B). After stress, mice significantly increased the time spent in the diestrus phase compared to control mice (Fig. 4C). Stress significantly elongated the diestrus phase, while other phases remained at the same length or shorter (Fig. 4C).

**Table 2:** Statistical information for Fig. 4

4A) Pre-stress	F(DFn, DFd)	p
Phase	F(3, 68) = 3.68	0.016
Stress	F(1, 68) = 0.02	0.88
Phase x stress	F(3, 68) = 1.67	0.18
4B) during stress		
Phase	F(3, 68) = 9.43	0.0001
Stress	F(1, 68) = 3.35e-19	0.99
Phase x stress	F(3, 68) = 8.17	0.0001
4C) After stress		
Phase	F(3, 68) = 6.63	0.0005
Stress	F(1, 68) = 2.01e-17	0.99
Phase x stress	F(3, 68) = 4.96	0.0036

### Stress mice become arrested in Diestrus phase

**Figure 5:**
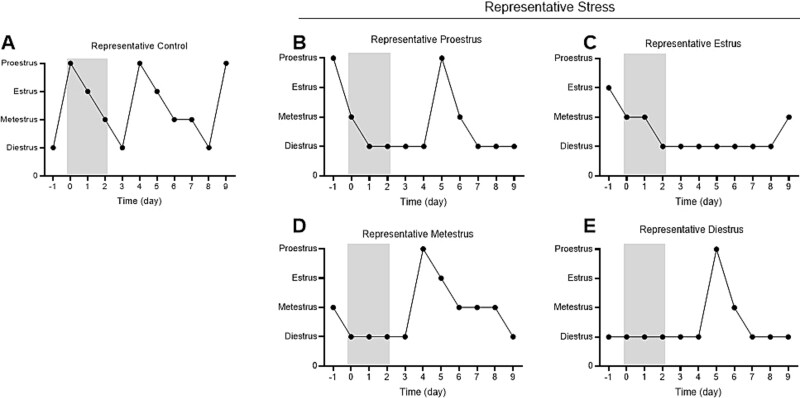
Representative graphs of daily phase tracking. Day 0 indicates the day the stress was initiated. Control (A) as the left graph, experimental as the right graphs (B-E). Stress period is represented by shaded rectangle; control mice remained in home cage during stress period.

To assess the effect of stress on estrous cyclicity, the temporal pattern of cycle phases was plotted to generate representative graphs for stressed and control mice. In the control condition, mice exhibited regular estrous cycles, progressing sequentially through each phase and completing two full cycles (Fig. 5A). In contrast, mice exposed to stress showed disrupted cycling, completing zero to one full cycle (Fig. 5B-E). Notably, stressed mice stayed in the diestrus phase, regardless of the phase they were in at the onset of stress (Fig. 5B-E).

## Discussion

This study examined how mild stress impacts the mouse estrous cycle using acute restraint stress. As fertility rates decline and women report stress at a higher level, identifying how stress impacts the female hormonal cycle becomes necessary (Driscoll and Hamilton 2025; APA, 2023). This study showed a causal link between mild stress and compromised female estrous cyclicity. We determined that restraint stress alters the mouse estrous cycle by lengthening the cycle and increasing the amount of time spent in the diestrus phase. In all stressed mice, the diestrus phase was rapidly approached and markedly long. This confirmed our hypothesis that mild stress dysregulates the mouse estrous cycle and demonstrates a direct causal relationship between stress and female mouse reproductive health. Our findings reinforce the idea that stress stops cycling (Vigil et al. 2022).

Our findings show that acute restraint stress altered the mouse estrous cycle through lengthening of the estrous cycle. The elongation of the estrous cycle occurred regardless of the phase the mice began stress in, as seen in Fig. 5. Because each mouse approached and was arrested in the diestrus phase, the data indicates that stress impaired estrous cycle regularity and specifically prolonged the diestrus phase. A lengthened diestrus phase could reinforce the idea that stress causes a state of lower neuronal signaling in the arcuate nucleus.

Studies have shown that acute psychosocial stress alters Kiss1 neuronal signaling which results in lowered Luteinizing Hormone (LH) levels due to increased RF-amide Related protein (Rfrp) gene expression (Yang et al. 2017). Rfrp acts as a Gonadotropin Inhibiting Hormone (GnIH) causing Gonadotropin Releasing Hormone (GnRH) suppression. Inhibition of the pulsatile frequency of GnRH leads to less production of Follicle Stimulating Hormone (FSH) and Luteinizing Hormone (LH) production (McCosh et al. 2022; Wagenmaker et al. 2009). Suppression of GnRH and LH pulses due to restraint (Yang et al. 2017) may explain that the cycle stalls in diestrus as a decrease in LH pulsation prevents the LH surge necessary for ovulation and the continued estrous cycle. While more experiments would be required to show that this mechanism is occurring in our mild stress model, it would explain the results observed in this study. By having dysregulated neuronal signaling, a female could be prevented from normal cycling, resulting in decreased fertility and impaired reproductive function.

## Conclusion

Our understanding of stress and the female body is still limited, and studies focusing on hormonal interplay are greatly needed to understand potential fertility implications. Further research on how acute restraint stress impacts fertility aspects such as ovulation, pregnancy, and pups per litter would increase our knowledge of the impact of stress on the female body. Animal models serve as an essential tool to determine the molecular mechanisms behind stress-related consequences to fertility.

## Supplementary Material

Supplementary_materials_kvag002

## Data Availability

Data available upon reasonable request.
