# Supplementary material for: Restraint stress prolongs Diestrus phase of mouse Estrous cycle
Source: Oxf Open Neurosci. 2026 Mar 18;5:kvag002. doi: 10.1093/oons/kvag002 (PMC13089459; doi:10.1093/oons/kvag002)
Supplement: Supplementary_materials_kvag002 [file supplementary_materials_kvag002.zip › Supplemental file for export.docx]

First round of review

Reviewer 1

The studies reported in this manuscript examined the effects of restraint stress on the mouse estrous cycle using a stress paradigm which may model more accurately day-to-day stress. To do this, the authors implemented a restraint stress procedure where mice were restrained for 2 hours a day across 3 days, and then collected lavage samples prior to, during, and for a number of days after the stress exposure. The authors then assessed the impact of stress on estrous cycle phases from the samples collected across time. The authors report that restraint stress not only disrupted the number of completed estrous cycles across post stress assessment days, but also elongated the diestrus phase compared with control animals. Furthermore, the authors report that no other phases were disrupted in this manner.

The findings reported in this manuscript provide insight on role that mild repeated stress could play in influencing fertility-related outcomes. The findings reported in this manuscript have clear and important translational implications. The experiments are well-designed, the approach is straightforward, and the manuscript is well-written. This reviewer has no issues with the authors’ interpretations of the reported data. I have only a few minor comments and suggestions.
In the Animals section of the Methods, there is no information provided about how the animals were obtained or where they came from. Were they ordered from an animal vendor? Or were they bred from the lab’s colony. If they were bred on campus, were litter effects taken into consideration when enrolling animals in groups? How was group assignment determined if this was the case (e.g., how many animals from one litter were assigned to a given group)? If they were acquired from an animal vendor, this should be stated and the vendor identified.
Under the Timeline section, it is stated that the control animals were “left alone during stress period in home room”. Were the animals placed into a separate holding area away from cagemates? Or does this mean that during this time there were simply no interactions with experimenters but also that they remained in their home cage with their cagemates?
For Table 1, the criteria for counting as “cyclic” that was given in the Statistical Analysis section defines a specific order of going through each phase across each cycle counted. This does not always align with the order of the phases for each of the defined cycles. For example subject F1 is initially going in order, then from November 13th to Nov 14 the animal goes from phase 4 to phase 2. This is still counted as being part of the cycle, though. Is this accurately done and what really needs to update is the description/criteria? Why wouldn’t November 14-18th be counted as a cycle for subject F1, based on the provided criteria? Why are the phases during November 11th-13th considered part of the first complete cycle for F1? There are similar issues with the cycles for F4.
Minor comment: The different panels of Figure 6 are identified by letter (e.g., Figure 6A) in the main text and also in the figure legend, however the letters associated with each panel are missing on the actual figure.
Minor comment: Figure 7 could benefit from also including letters to label each of the representative graphs or groupings of the graphs. A present, one general reference to Figure 7 is used to refer to the different graphs within this figure. Additionally, the figure legend for this figure states at the end that “control mice were not moved during this period”. As with the previous comment related to the handling of the mice during this phase of the study, the reader may benefit from additional detail to the wording for clarity.

Reviewer 2

This manuscript examines the effects of mild, repeated restraint stress on reproductive cyclicity using adult female C57BL/6J mice as model system. Estrous cycling was assessed via daily vaginal lavaging before, during, and after exposure to two hours of restraint, per day, for three consecutive days. Compared to control animals, stressed-exposed mice had significantly fewer estrous cycles and displayed a marked increase in overall cycle length. The researchers find that this disruption was driven primarily by a pronounced prolongation of the diestrus phase, with many stressed mice becoming arrested in diestrus regardless of the phase at stress onset. These alterations extended into the post-stress period, indicating lasting effects on reproductive cyclicity. Overall, the study provides experimental evidence that mild stressors are sufficient to perturb estrous cycle dynamics. The manuscript is well organized, the experimental approach is appropriate, and the results are clearly presented; only minor revisions are required to improve clarity and framing.

Significance
This study addresses an important and timely question on the impact of everyday stressors on female reproductive health. It demonstrates that mild stress can induce sustained disruptions in estrous cycling, through prolongation of the diestrus phase. These findings offer mechanistic insight into how mild stress may contribute to reduced fertility. The work strengthens the translational relevance of stress-based animal models and highlights the importance of mild stress exposures on reproductive outcomes in females.

Minor Revisions
1. Please clarify if animals were single-housed or group-housed during the stress exposure period and whether this housing condition continued into the post-stress phase. Given the known influence of group-housing on stress responsivity and reproductive cycling, this information would aid in interpreting the findings.

2. Could the authors provide a brief justification for assigning a value of 10 days to non-cycling restrained animals (data points from Table 1 and Figure 4). Clarifying the rationale (e.g., maximum observation window) would increase future reproducibility.

3. Please clarify if Figure 3 reflects post-stress lavage data collected over 7 days, or combined stress & post-stress lavages spanning 10 days.

Other Minor Points:
• Page 3, Line 64: Capitalize the “L” in mL.
• Figure 2: Change “Samples” to lowercase (samples).
• Figure 2: Replace “2hr” with “2 h” for consistency.
• Figure 3 (graph): Replace numerical p-value text (p < 0.0001) with asterisks (**) to denote statistical significance, consistent with other figure conventions.
• Figure 3 (caption): Please confirm and correct the sample size to n = 10 for stress-exposed animals (currently listed as 11).
• Figure 4 (caption): Change “T-test” to lowercase (t-test).
• Figure 5 (caption): Remove the equals sign “=” preceding p < 0.001.
• Figure 6:
o Add letter labels (A, B, C) to individual graphs to match the Results section descriptions.
o Remove “ns” labels across graphs, as these may be redundant given the statistical reporting.
• Figure 7: Adjust line colors to improve contrast and readability, as the current color scheme is difficult to distinguish.
• Discussion section: Double-check in-text citations, as several instances are missing a comma following “et al.” (et al.,)
• Page 9, Line 185: Remove the ellipsis (“…”) following the word litter.

| **Preview (OXFNSC-2025-005)** |
| --- |
| \| **From:** \| aizquie@psych.ucla.edu \| \| --- \| --- \| \| **To:** \| laumetge@msu.edu \| \| **CC:** \|  \| \| **BCC:** \| aizquie@psych.ucla.edu \| \| **Subject:** \| Oxford Open Neuroscience - Decision on Manuscript ID OXFNSC-2025-005 \| \| **Body:** \| 22-Jan-2026  Dear Dr. Laumet,  Manuscript ID OXFNSC-2025-005 entitled "Restraint Stress Prolongs Diestrus Phase of Mouse Estrous Cycle" which you submitted to the Oxford Open Neuroscience, has been reviewed.  Reviewer comments are included at the bottom of this letter.  The reviewers have suggested some minor revisions to your manuscript. Therefore, I invite you to respond to the reviewers' comments and revise your manuscript within the next 30 days of todays date. In addition to the reviewers' comments, I have some formatting suggestions to improve data visualization. I suggest to combine current Figures 3, 4, and 5 into 1 multi-paneled Figure 3 (panels A, B, and C), format Table 1 to match the format of Table 2 (no vertical lines, no color, just shading, standard table format), and label current Figure 6 with panels A, B and C (would be new Figure 4). Current Figure 7 (would be new Figure 5) should also be improved in its resolution as some of the figures are blurry.  Please note that this journal operates with transparent peer review. This means that if your submission is accepted for publication, the full peer review history of your article will publish online alongside your article. This includes reviewer comments, editor decision letters, and your author responses.  To revise your manuscript, log into https://mc.manuscriptcentral.com/oxfnsc and enter your Author Centre, where you will find your manuscript title listed under "Manuscripts with Decisions."  Under "Actions," click on "Create a Revision."  Your manuscript number has been appended to denote a revision.  You may also click the below link to start the revision process (or continue the process if you have already started your revision) for your manuscript. If you use the below link you will not be required to login to ScholarOne Manuscripts.  *** PLEASE NOTE: This is a two-step process. After clicking on the link, you will be directed to a webpage to confirm. ***  https://mc.manuscriptcentral.com/oxfnsc?URL_MASK=357039fd41254b2ba479e6de2e9cb4e2   You will be unable to make your revisions on the originally submitted version of the manuscript.  Instead, revise your manuscript using a word processing program and save it on your computer.  Please also highlight the changes to your manuscript within the document by using the track changes mode in MS Word or by using bold or colored text.  Once the revised manuscript is prepared, you can upload it and submit it through your Author Centre.  When submitting your revised manuscript, you will be able to respond to the comments made by the reviewer(s) in the space provided.  You can use this space to document any changes you make to the original manuscript.  In order to expedite the processing of the revised manuscript, please be as specific as possible in your response to the reviewer(s).  IMPORTANT:  Your original files are available to you when you upload your revised manuscript.  Please delete any redundant files before completing the submission.  Because we are trying to facilitate timely publication of manuscripts submitted to the Oxford Open Neuroscience, your revised manuscript should be uploaded within the next 30 days from todays date.  If it is not possible for you to submit your revision in a reasonable amount of time, we may have to consider your paper as a new submission.  Once again, thank you for submitting your manuscript to the Oxford Open Neuroscience and I look forward to receiving your revision.  Sincerely, Prof. Alicia Izquierdo Senior Editor, Oxford Open Neuroscience aizquie@psych.ucla.edu   Reviewer: 1  Comments to the Author The studies reported in this manuscript examined the effects of restraint stress on the mouse estrous cycle using a stress paradigm which may model more accurately day-to-day stress. To do this, the authors implemented a restraint stress procedure where mice were restrained for 2 hours a day across 3 days, and then collected lavage samples prior to, during, and for a number of days after the stress exposure. The authors then assessed  the impact of stress on estrous cycle phases from the samples collected across time. The authors report that restraint stress not only disrupted the number of completed estrous cycles across post stress assessment days, but also elongated the diestrus phase compared with control animals. Furthermore, the authors report that no other phases were disrupted in this manner.  The findings reported in this manuscript provide insight on the role that mild repeated stress could play in influencing fertility-related outcomes. The findings reported in this manuscript have clear and important translational implications. The experiments are well-designed, the approach is straightforward, and the manuscript is well-written. This reviewer has no issues with the authors’ interpretations of the reported data. I have only a few minor comments and suggestions. In the Animals section of the Methods, there is no information provided about how the animals were obtained or where they came from. Were they ordered from an animal vendor? Or were they bred from the lab’s colony? If they were bred on campus, were litter effects taken into consideration when enrolling animals in groups? How was group assignment determined if this was the case (e.g., how many animals from one litter were assigned to a given group)? If they were acquired from an animal vendor, this should be stated and the vendor identified. Under the Timeline section, it is stated that the control animals were “left alone during stress period in home room”. Were the animals placed into a separate holding area away from cagemates? Or does this mean that during this time there were simply no interactions with experimenters but also that the animals remained in their home cage with their cagemates? For Table 1, the criteria for counting as “cyclic” that was given in the Statistical Analysis section defines a specific order of going through each phase across each cycle counted. This does not always align with the order of the phases for each of the defined cycles. For example subject F1 is initially going in order, then from November 13th to Nov 14 the animal goes from phase 4 to phase 2. This is still counted as being part of the cycle, though. Is this accurate and what really needs to update is the description/criteria? Why wouldn’t November 14-18th be counted as a cycle for subject F1, based on the provided criteria? Why are the phases during November 11th-13th considered part of the first complete cycle for F1? There are similar issues with the cycles for F4. Minor comment: The different panels of Figure 6 are identified by letter (e.g., Figure 6A) in the main text and also in the figure legend, however the letters associated with each panel are missing on the actual figure. Minor comment: Figure 7 could benefit from also including letters to label each of the representative graphs or groupings of the graphs. A present, one general reference to Figure 7 is used to refer to the different graphs within this figure. Additionally, the figure legend for this figure states at the end that “control mice were not moved during this period”. As with the previous comment related to the handling of the mice during this phase of the study, the reader may benefit from additional detail to the wording for clarity.   Reviewer: 2  Comments to the Author This manuscript examines the effects of mild, repeated restraint stress on reproductive cyclicity using adult female C57BL/6J mice as model system. Estrous cycling was assessed via daily vaginal lavaging before, during, and after exposure to two hours of restraint, per day, for three consecutive days. Compared to control animals, stressed-exposed mice had significantly fewer estrous cycles and displayed a marked increase in overall cycle length. The researchers find that this disruption was driven primarily by a pronounced prolongation of the diestrus phase, with many stressed mice becoming arrested in diestrus regardless of the phase at stress onset. These alterations extended into the post-stress period, indicating lasting effects on reproductive cyclicity. Overall, the study provides experimental evidence that mild stressors are sufficient to perturb estrous cycle dynamics. The manuscript is well organized, the experimental approach is appropriate, and the results are clearly presented; only minor revisions are required to improve clarity and framing.  Significance This study addresses an important and timely question on the impact of everyday stressors on female reproductive health. It demonstrates that mild stress can induce sustained disruptions in estrous cycling, through prolongation of the diestrus phase. These findings offer mechanistic insight into how mild stress may contribute to reduced fertility. The work strengthens the translational relevance of stress-based animal models and highlights the importance of mild stress exposures on reproductive outcomes in females.  Minor Revisions 1. Please clarify if animals were single-housed or group-housed during the stress exposure period and whether this housing condition continued into the post-stress phase. Given the known influence of group-housing on stress responsivity and reproductive cycling, this information would aid in interpreting the findings.  2. Could the authors provide a brief justification for assigning a value of 10 days to non-cycling restrained animals (data points from Table 1 and Figure 4). Clarifying the rationale (e.g., maximum observation window) would increase future reproducibility.  3. Please clarify if Figure 3 reflects post-stress lavage data collected over 7 days, or combined stress & post-stress lavages spanning 10 days.  Other Minor Points: • Page 3, Line 64: Capitalize the “L” in mL. • Figure 2: Change “Samples” to lowercase (samples). • Figure 2: Replace “2hr” with “2 h” for consistency. • Figure 3 (graph): Replace numerical p-value text (p < 0.0001) with asterisks (**) to denote statistical significance, consistent with other figure conventions. • Figure 3 (caption): Please confirm and correct the sample size to n = 10 for stress-exposed animals (currently listed as 11). • Figure 4 (caption): Change “T-test” to lowercase (t-test). • Figure 5 (caption): Remove the equals sign “=” preceding p < 0.001. • Figure 6: o Add letter labels (A, B, C) to individual graphs to match the Results section descriptions. o Remove “ns” labels across graphs, as these may be redundant given the statistical reporting. • Figure 7: Adjust line colors to improve contrast and readability, as the current color scheme is difficult to distinguish. • Discussion section: Double-check in-text citations, as several instances are missing a comma following “et al.” (et al.,) • Page 9, Line 185: Remove the ellipsis (“…”) following the word litter.  Associate Editor Comments to the Author: Thank you for submitting your manuscript to Oxford Open Neuroscience.  I have completed my evaluation of your manuscript. It will need Minor revisions in order to be considered for publication. When revising your manuscript please outline every change made in response to the reviewers' comments, and provide suitable rebuttals for any comments not addressed. Please note that your revised submission may need to be re-reviewed. \| \| \| **Date Sent:** \| 22-Jan-2026 \|  \| |

Response to reviewers

**Editor**. I have some formatting suggestions to improve data visualization. I suggest to combine current Figures 3, 4, and 5 into 1 multi-paneled Figure 3 (panels A, B, and C), format Table 1 to match the format of Table 2 (no vertical lines, no color, just shading, standard table format), and label current Figure 6 with panels A, B and C (would be new Figure 4). Current Figure 7 (would be new Figure 5) should also be improved in its resolution as some of the figures are blurry.

Thank you for your suggestion! We combined Figures 3-5, reformatted Table 1, update the labeling of revised Figures 5,6, and improved resolution.

Please see below our point-by-point answers to the reviewers in purple. We appreciate the reviewer’s comments that have helped us to strengthen our manuscript. Modifications in the manuscript are marked in purple.

Please note that this journal operates with transparent peer review. This means that if your submission is accepted for publication, the full peer review history of your article will publish online alongside your article. This includes reviewer comments, editor decision letters, and your author responses. We concur with the journal reviewing policy.

**Reviewer #1**. The studies reported in this manuscript examined the effects of restraint stress on the mouse estrous cycle using a stress paradigm which may model more accurately day-to-day stress. To do this, the authors implemented a restraint stress procedure where mice were restrained for 2 hours a day across 3 days, and then collected lavage samples prior to, during, and for a number of days after the stress exposure. The authors then assessed  the impact of stress on estrous cycle phases from the samples collected across time. The authors report that restraint stress not only disrupted the number of completed estrous cycles across post stress assessment days, but also elongated the diestrus phase compared with control animals. Furthermore, the authors report that no other phases were disrupted in this manner.

The findings reported in this manuscript provide insight on the role that mild repeated stress could play in influencing fertility-related outcomes. The findings reported in this manuscript have clear and important translational implications. The experiments are well-designed, the approach is straightforward, and the manuscript is well-written. This reviewer has no issues with the authors’ interpretations of the reported data. I have only a few minor comments and suggestions.

We thank Reviewer #1 for their thorough assessment and for acknowledging the rigor of experimental design and the clarity.

In the Animals section of the Methods, there is no information provided about how the animals were obtained or where they came from. Were they ordered from an animal vendor? Or were they bred from the lab’s colony? If they were bred on campus, were litter effects taken into consideration when enrolling animals in groups? How was group assignment determined if this was the case (e.g., how many animals from one litter were assigned to a given group)? If they were acquired from an animal vendor, this should be stated and the vendor identified.

We thank Reviewer #1 for raising this important point and apologize for the lack of clarity in the original Methods section. The breeder mice were originally obtained from **The Jackson Laboratory** and subsequently bred in our laboratory colony. To minimize potential litter effects, each experimental cage consisted of animals from a single litter (3–5 female mice per cage). Cages, rather than individual animals, were used as the unit of randomization: cages were randomly assigned to the control condition or to the stress condition. This information has now been added to the Methods.

Under the Timeline section, it is stated that the control animals were “left alone during stress period in home room”. Were the animals placed into a separate holding area away from cagemates? Or does this mean that during this time there were simply no interactions with experimenters but also that the animals remained in their home cage with their cagemates?

We thank the reviewer for the opportunity to clarify this point. During the stress period, control animals had no interactions with experimenters and remained undisturbed in their home cages with their cagemates in the home room. They were not moved to a separate holding area. This clarification has now been added to the Methods.

For Table 1, the criteria for counting as “cyclic” that was given in the Statistical Analysis section defines a specific order of going through each phase across each cycle counted. This does not always align with the order of the phases for each of the defined cycles. For example subject F1 is initially going in order, then from November 13th to Nov 14 the animal goes from phase 4 to phase 2. This is still counted as being part of the cycle, though. Is this accurate and what really needs to update is the description/criteria? Why wouldn’t November 14-18th be counted as a cycle for subject F1, based on the provided criteria? Why are the phases during November 11th-13th considered part of the first complete cycle for F1? There are similar issues with the cycles for F4.

We thank the reviewer for pointing out this source of confusion. We agree that the original description of the criteria for defining a “cycle” lacked sufficient clarity and could be misinterpreted.

For subject F1, cycle counting was initiated on the first day of sampling (November 11th) and continued until all four estrous stages were observed in the correct order at least once. Because vaginal lavage was not performed on November 10th, we could not infer the estrous stage preceding November 11th. As a result, we conservatively defined the first complete cycle for F1 as spanning November 11th–18th, which represents the first observation window in which all four stages in order were documented. Although the animal transitioned from stage 4 to stage 2 between November 13th and 14th, this transition did not initiate a new cycle, as the criteria required completion of a full ordered sequence rather than restarting the count mid-sequence.

The reviewer is correct in noting an error in the cycle assignment for subject F4. Upon re-evaluation, F4 completed one cycle between November 11th–14th and a second cycle between November 15th–22^nd^ (Table 1 corrected). Importantly, correcting this mistake does not alter the statistical analyses or conclusions of the study.

To address these issues, we have revised the Methods section to more clearly define how estrous cyclicity and cycle boundaries were determined. The updated text now explicitly states that a mouse was classified as cyclic if it progressed through all four estrous stages in the correct order at least once during the observation period, that repeated stages were permitted, and that cycle counting began on the first day of observation due to the absence of prior lavage data. These clarifications resolve the discrepancies identified by the reviewer.

Minor comment: The different panels of Figure 6 are identified by letter (e.g., Figure 6A) in the main text and also in the figure legend, however the letters associated with each panel are missing on the actual figure. We corrected it.
Minor comment: Figure 7 could benefit from also including letters to label each of the representative graphs or groupings of the graphs. A present, one general reference to Figure 7 is used to refer to the different graphs within this figure. We added the letter to label the panels. Additionally, the figure legend for this figure states at the end that “control mice were not moved during this period”. As with the previous comment related to the handling of the mice during this phase of the study, the reader may benefit from additional detail to the wording for clarity. We clarified it accordingly to the response #2 above.

**Reviewer #2**. This manuscript examines the effects of mild, repeated restraint stress on reproductive cyclicity using adult female C57BL/6J mice as model system. Estrous cycling was assessed via daily vaginal lavaging before, during, and after exposure to two hours of restraint, per day, for three consecutive days. Compared to control animals, stressed-exposed mice had significantly fewer estrous cycles and displayed a marked increase in overall cycle length. The researchers find that this disruption was driven primarily by a pronounced prolongation of the diestrus phase, with many stressed mice becoming arrested in diestrus regardless of the phase at stress onset. These alterations extended into the post-stress period, indicating lasting effects on reproductive cyclicity. Overall, the study provides experimental evidence that mild stressors are sufficient to perturb estrous cycle dynamics. The manuscript is well organized, the experimental approach is appropriate, and the results are clearly presented; only minor revisions are required to improve clarity and framing.

We thank Reviewer #2 for their positive evaluation of our work and for recognizing its significance, rigor, and translational relevance.

Significance
This study addresses an important and timely question on the impact of everyday stressors on female reproductive health. It demonstrates that mild stress can induce sustained disruptions in estrous cycling, through prolongation of the diestrus phase. These findings offer mechanistic insight into how mild stress may contribute to reduced fertility. The work strengthens the translational relevance of stress-based animal models and highlights the importance of mild stress exposures on reproductive outcomes in females.

Minor Revisions
1. Please clarify if animals were single-housed or group-housed during the stress exposure period and whether this housing condition continued into the post-stress phase. Given the known influence of group-housing on stress responsivity and reproductive cycling, this information would aid in interpreting the findings.

Animals were group-housed throughout the experiment, except during the restraint stress sessions, when mice were individually restrained in conical tubes. Information will be added to the methods.

2. Could the authors provide a brief justification for assigning a value of 10 days to non-cycling restrained animals (data points from Table 1 and Figure 4). Clarifying the rationale (e.g., maximum observation window) would increase future reproducibility.

We thank the reviewer for this question. The value of 10 days corresponds to the predefined maximum observation window used in this study. Animals that did not complete a full estrous cycle within this time frame were classified as non-cycling. This arbitrary observation time allowed consistent comparability across experimental groups. We have clarified this rationale in the Methods section to improve transparency and reproducibility.

3. Please clarify if Figure 3 reflects post-stress lavage data collected over 7 days, or combined stress & post-stress lavages spanning 10 days.

Figure 3 combines “stress” (same days as restrain paradigm) and post-stress lavage data spanning 10 days. We clarified the Figure legend.

Other Minor Points: All have been corrected.
• Page 3, Line 64: Capitalize the “L” in mL.
• Figure 2: Change “Samples” to lowercase (samples).
• Figure 2: Replace “2hr” with “2 h” for consistency.
• Figure 3 (graph): Replace numerical p-value text (p < 0.0001) with asterisks (**) to denote statistical significance, consistent with other figure conventions.
• Figure 3 (caption): Please confirm and correct the sample size to n = 10 for stress-exposed animals (currently listed as 11).
• Figure 4 (caption): Change “T-test” to lowercase (t-test).
• Figure 5 (caption): Remove the equals sign “=” preceding p < 0.001.
• Figure 6:
o Add letter labels (A, B, C) to individual graphs to match the Results section descriptions.
o Remove “ns” labels across graphs, as these may be redundant given the statistical reporting.
• Figure 7: Adjust line colors to improve contrast and readability, as the current color scheme is difficult to distinguish.
• Discussion section: Double-check in-text citations, as several instances are missing a comma following “et al.” (et al.,)
• Page 9, Line 185: Remove the ellipsis (“…”) following the word litter.

Second round of review

Reviewer 1

The authors have appropriately addressed this reviewer's comments/suggestions/concerns.

Reviewer 2

All of my comments were addressed – no further revisions needed. I recommend this manuscript for publication.

| **Preview (OXFNSC-2025-005.R1)** |
| --- |
| \| **From:** \| aizquie@psych.ucla.edu \| \| --- \| --- \| \| **To:** \| laumetge@msu.edu \| \| **CC:** \| aizquie@psych.ucla.edu \| \| **BCC:** \| sara.yannytillar@oup.com \| \| **Subject:** \| Oxford Open Neuroscience - Decision on Manuscript ID OXFNSC-2025-005.R1 \| \| **Body:** \| 02-Mar-2026  Dear Dr. Laumet,  It is a pleasure to accept your revised manuscript entitled "Restraint Stress Prolongs Diestrus Phase of Mouse Estrous Cycle" in its current form for publication in the Oxford Open Neuroscience. The comments of the reviewer(s) who reviewed your manuscript are included at the foot of this letter.  Please note that this journal operates with transparent peer review. This means that  the full peer review history of your article will publish online alongside your article. This includes reviewer comments, editor decision letters, and your author responses.  Next steps You will receive an email from no-reply@scipris.com within roughly one week. This is your invitation to sign up for an account with SciPris, Oxford University Press’ author portal hosted by Aptara. You will need to create an account if you do not already hold one. Please register or log into your account and follow the online instructions which will guide you through signing your licence and paying the APC. The email and the portal have clearly signposted support options if you need any help during this process.  Please note that SciPris is a completely different system from ScholarOne, so your credentials to submit your manuscript here will not work there. Once you’ve created a SciPris account, you will be able to use it whenever you publish with Oxford Open Neuroscience or any OUP journal. Please note that OUP will only ever request payment for applicable fees be made via SciPris or to an OUP bank account. If you ever have concerns about the legitimacy of a request, please do not hesitate to contact a customer services agent via the SciPris portal or directly via oupsupport@scipris.com.  Thank you for your fine contribution.  On behalf of the Editors of the Oxford Open Neuroscience, we look forward to your continued contributions to the Journal.  Sincerely, Prof. Alicia Izquierdo Senior Editor, Oxford Open Neuroscience aizquie@psych.ucla.edu   Reviewer: 1  Comments to the Author The authors have appropriately addressed this reviewer's comments/suggestions/concerns.  Reviewer: 2  Comments to the Author All of my comments were addressed – no further revisions needed. I recommend this manuscript for publication.  Associate Editor Comments to the Author: Please ensure Figure 4 caption statistics are associated with revised figure number panels 4A-C (not 6A-C). \| \| \| **Date Sent:** \| 02-Mar-2026 \|  \| |
